# *QuickStats:* Rate* of Unintentional Traumatic Brain Injury (TBI)–Related Deaths^†^ Among Persons Aged ≤24 Years, by Age Group — National Vital Statistics System, United States, 1999–2018

**DOI:** 10.15585/mmwr.mm6941a5

**Published:** 2020-10-16

**Figure:**
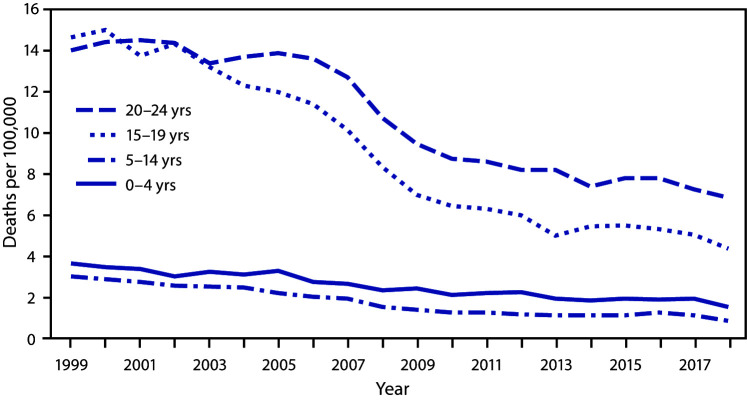
From 1999 to 2018, death rates for unintentional TBI among persons aged ≤24 years declined across all age groups. During the 20-year period, TBI-related death rates declined from 3.7 per 100,000 to 1.5 among children aged 0–4 years, from 3.0 to 0.9 for children and adolescents aged 5–14 years, from 14.7 to 4.4 for adolescents and young adults aged 15–19 years, and from 14.1 to 6.9 for young adults aged 20–24 years. For most of the period, rates were highest for persons aged 20–24 years followed by those aged 15–19, 0–4, and 5–14 years.

**Source:** National Center for Health Statistics, National Vital Statistics System, Mortality Data. https://www.cdc.gov/nchs/deaths.htm.

